# Perceptual Mechanisms of Visual Hallucinations and Illusions in Psychosis

**DOI:** 10.20900/jpbs.20200020

**Published:** 2020-08-21

**Authors:** Samuel D. Klein, Cheryl A. Olman, Scott R. Sponheim

**Affiliations:** 1 Clinical Science and Psychopathology Research Program, University of Minnesota-Twin Cities, 75 East River Road, Minneapolis, MN 55455, USA; 2 Department of Psychology, University of Minnesota-Twin Cities, 75 East River Road, Minneapolis, MN 55455, USA; 3 Center for Magnetic Resonance Research, University of Minnesota-Twin Cities, 2021 6th St SE, Minneapolis, MN 55455, USA; 4 Minneapolis Veterans Affairs Health Care System, 1 Veterans Dr, Minneapolis, MN 55417, USA; 5 Department of Psychiatry and Behavioral Science, University of Minnesota, 606 24th Ave S, Minneapolis, MN 55454, USA

**Keywords:** psychosis, fMRI, visual perception, MEG, cortical source signaling, endophenotype

## Abstract

Psychosis has been associated with neural anomalies across a number of brain regions and cortical networks. Nevertheless, the exact pathophysiology of the disorder remains unclear. Aberrant visual perceptions such as hallucinations are evident in psychosis, while the occurrence of visual distortions is elevated in individuals with genetic liability for psychosis. The overall goals of this project are to: (1) use psychophysical tasks and neuroimaging to characterize deficits in visual perception; (2) acquire a mechanistic understanding of these deficits through development and validation of a computational model; and (3) determine if said mechanisms mark genetic liability for psychosis. Visual tasks tapping both low- and high-level visual processing are being completed as individuals with psychotic disorders (IPD), first-degree biological siblings of IPDs (SibIPDs) and healthy controls (HCs) undergo 248-channel magneto-encephalography (MEG) recordings followed by 7 Tesla functional magnetic resonance imaging (MRI). By deriving cortical source signals from MEG and MRI data, we will characterize the timing, location and coordination of neural processes. We hypothesize that IPDs prone to visual hallucinations will exhibit deviant functions within early visual cortex, and that aberrant contextual influences on visual perception will involve higher-level visual cortical regions and be associated with visual hallucinations. SibIPDs who experience visual distortions—but not hallucinations—are hypothesized to exhibit deficits in higher-order visual processing reflected in abnormal inter-regional neural synchronization. We hope the results lead to the development of targeted interventions for psychotic disorders, as well as identify useful biomarkers for aberrant neural functions that give rise to psychosis.

## SIGNIFICANCE

At present, abnormal neural functions that give rise to psychotic experiences remain poorly understood. As a result, present pharmacological and behavioral interventions remain unguided by etiologic mechanisms [1,2]. A promising avenue of research for revealing the neural origins of psychosis lies in examining visual perceptual anomalies in individuals with psychotic disorders. Visual hallucinations are common in individuals with psychosis [3], while visual distortions and altered illusions are evident in those with genetic liability for psychosis (i.e., first-degree biological relatives; [4]). Generally, the incidence of visual hallucinations is estimated to be between 25–30 percent [3]. A recent report nonetheless found that more than 50% of patients with schizophrenia in a US sample experienced visual hallucinations [5]. Critically, these findings generalize across countries: a recent systematic review found that visual hallucinations were experienced by 39.1% of a sample of 1080 patients with schizophrenia across seven countries in Europe, Asia and Africa [6]. In addition, visual hallucinations are associated with more severe psychotic symptoms, and poorer patient prognoses [3,7,8].

Less severe aberrations are often described as visual distortions. Visual distortions are more common than visual hallucinations [9]; their incidence is estimated to be at least double that of hallucinations [10]. Although visual processing deficits have been well documented in patients with schizophrenia [11–14], they are also seen across the psychosis spectrum—they have been documented in individuals at Ultra High Risk for conversion to a psychotic disorder [15,16] and in individuals with schizotypal personality disorder [17–19]. Moreover, it appears that relatives of patients with a psychotic disorder likewise display deficits in visual processing [20–22]. Thus, the study of aberrant visual perception spans diagnostic and genetic aspects of the psychosis spectrum.

The purpose of the proposed work is to combine measures of scene segmentation and surround suppression behaviors as a measure of perceptual organization in schizophrenia. Surround suppression tasks are thought to (1) involve gain control mechanisms in early visual cortex [23]; and (2) rely on inhibitory GABAergic neurotransmission [24]. The process of scene segmentation (alternatively conceptualized as perceptual grouping) involves parsing of visual scenes into coherent patterns (i.e., texture boundaries) and objects (i.e., surface segregation; [25]). It appears that processes involved in the detections of texture boundaries involve feedforward signals from V1 to higher visual areas in parietal and temporal cortices, while surface segregation relies on feedback connections originating in temporal areas and extending to V1 [26]. Patients with psychotic disorders consistently demonstrate deficits in early visual processing [27–29]. Examining the upstream and downstream connections from early visual cortex could elucidate the neural mechanisms underlying a robust finding in the study of psychotic disorders.

It has been suggested that early visual processing is partially dependent on local gain control mechanisms [30,31], coupled with long-range inputs from higher areas along the visual pathway [32]. Therefore, “higher-order” visual areas provide feedback inputs to local gain control mechanisms [33]. It appears that both local and long-range influences— coupled with feedforward and feedback interactions between levels of visual processing—determine the accuracy of visual percepts [34]. Divisive normalization has been proposed as a cortical computation to explain a neuron’s response as determined by a driving afferent input and divisive input from a group of neurons in the same network [35]. Divisive gain control—a subset of divisive normalization—has been successfully applied to model neuronal properties in early visual cortex [36] encoding reward value in decision-making circuits [37], as well as multisensory integration [38].

**Figure 1.**
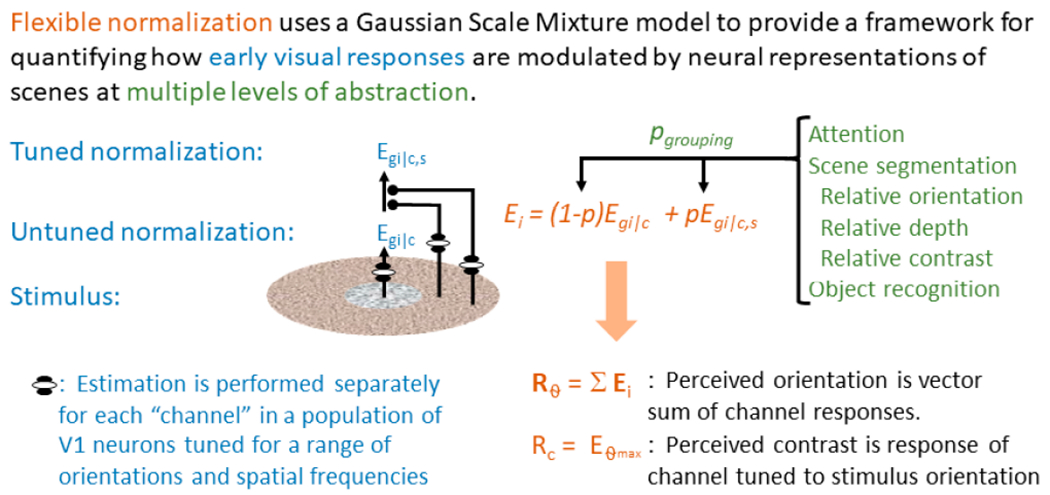
Expanded model originally proposed by Schwartz and colleagues and adapted in our previous work [41] to describe the influence of scene segmentation cues on the tilt illusion in HCs. The proposed model accounts for perceived contrast in a range of laboratory and naturalistic stimuli, as well as the interaction of perceived contrast with higher-level scene segmentation cues such as directed spatial attention and object recognition. This model lets us characterize visual perceptual abnormalities in terms of specific alterations to local inhibitory responses and local and long-range excitatory response.

Schwartz and colleagues [39] found that by revising a Gaussian Scale Mixture model (GSM) to account for the probability that a center and surround are part of the same visual object, one can closely approximate behavioral data from the tilt illusion. Given that GSM models are well-suited to modeling statistics in natural images [40], they may also serve to explain reduced suppressive effects induced by psychophysical laboratory paradigms in patients with schizophrenia [13]. By extending the model developed by Schwartz and colleagues [39] to generalize across multiple scene segmentation cues [41], one can compare the model parameters reflecting local and long-range inhibition to alterations in visual perception (see Figure 1).

Herein, we propose to examine the neural mechanisms underlying visual hallucinations and distortions in individuals with a psychotic disorder (IPDs) and first-degree biological siblings of individuals with a psychotic disorder (SibIPDs) and healthy controls (HC). The cortical networks underlying vision constitute some of the best-understood mechanisms within cognitive neuroscience—nearly a third of human cortex is devoted to visual processing [42]. Present theories suggest that early abnormalities in visual perception trigger a neural cascade leading to downstream alterations in visual experience in psychosis. Yet, the nature of how basic and complex visual functions give rise to aberrant visual perception in psychosis remains unclear. *The primary goals of the proposed work are to use psychophysical tasks and neuroimaging techniques to (1) precisely characterize behavioral and neural abnormalities in individuals with psychotic disorders during visual perception; (2) acquire a mechanistic understanding of these abnormalities through development and validation of a computational model; and (3) determine if said mechanisms mark genetic liability for psychosis (i.e., constitute a biomarker)*.

## SPECIFIC AIMS AND HYPOTHESES

By studying individuals that vary along the psychosis spectrum (e.g., schizophrenia, bipolar disorder with psychotic features, schizoaffective disorder), as well as individuals with intermediate clinical phenotypes (i.e., SibIPDs), we can determine how dimensional variation along the psychosis spectrum differentially influences the manifestation of visual hallucinations and visual distortions/illusions. The proposed work has the following specific aims:

### *Aim 1*: Assess the impact of scene segmentation cues on early visual processes in IPDs, their unaffected biological siblings (SibIPDs), and healthy controls (HCs), and determine how lab-based task performance predicts self-reported visual misperceptions.

H1: Individuals prone to visual hallucinations have reduced gain control in neural circuits of early visual cortex (V1, V2), which will be reflected by *improved* accuracy on a surround suppression task (i.e., reduced suppression); H2: Reduced intracortical connectivity within visual cortex (e.g., connectivity between V1 and V2), will be reflected in aberrant detection of co-linearity between visual elements and poor suppression of irrelevant elements of a visual scene; H3: Reduced activity in more anterior visual brain regions (LOC/fusiform, frontal cortex) and early visual cortex, will be characterized by deviant object identification due to weak use of high-level object templates to identify relevant targets in ambiguous scenes.

### *Aim 2*: Determine whether variation in self-reported visual hallucinations and illusions is associated with the location, timing and synchronization of neural responses in IPDs, SibIPDs, and HCs during perceptual tasks.

H4: IPDs prone to visual hallucinations will exhibit stable neural abnormalities in early visual cortex (V1, V2), and will be associated with errors in processing visual elements in simple scenes; H5: Visual hallucinations result in IPDs when long-range influences from “higher-order” brain regions (LOC/fusiform, prefrontal cortex) visual percepts are deviant, and will be reflected by anomalous inter-regional synchronization within theta/gamma frequencies; H6: SibIPDs prone to anomalous visual distortions will share this stable decrement in long-range influences on visual perception reflected in anomalous inter-regional synchronization in theta/gamma frequencies.

### *Aim 3*: Test whether a “flexible normalization” model can capture abnormal gain control in early visual responses and abnormal coordination between early visual areas with scene-based or goal-directed signals from other brain regions.

H7: Reduced surround suppression in IPDs (i.e., failure of local contextual modulation) can be characterized as a reduction in local gain control strength; there will be no contribution of intra-regional coordination; H8) Deficits in attention regulation and perception of complex scenes in IPDs and SibIPDs cannot be explained merely by reduced gain control and require an additional term describing reduced efficacy of long-range (intra-regional) projections.

## INNOVATION

The proposed work has several notable innovations. First, the assessment of early visual processing through behavioral task performance and attentional manipulations in the same individuals within a single study will elucidate the proportional influences of local and long-range mechanisms on self-reported visual hallucinations and distortions. Second, most visual psychophysical tasks employ an adaptive staircase procedure in order to individualize difficulty level to equate performance across subjects; doing so eliminates the generalized deficit confound that would be expected in IPDs (and to some extent SibIPDs), thereby equating true score variance across tasks. Third, combined use of 248-channel MEG and 7T fMRI to derive cortical source signals for retinotopically mapped areas of striate cortex will optimize both spatial and temporal resolution allowing for dynamic comparisons of neural responses across levels of visual processing. Fourth, quantitative modeling will parameterize the determinants of subjects’ visual percepts during specialized psychophysical tasks and allow integration of experimental findings across tasks, groups, and modalities. Fifth, MEG-derived cortical source signals will allow investigation of the interdependence between theta and gamma frequencies in occipital and other cortices during visual processing. Sixth, use of the Reduced Interference Distribution (RID) time frequency (TF) transform will yield a precise characterization of the timing of select frequency band activity [43,44]; this method avoids energy loss and trade-off between time and frequency resolution that occurs with traditional wavelet analysis [45]. Seventh, the inclusion of IPDs beyond traditional diagnostic boundaries of schizophrenia, as well as SibIPDs—some of whom experience visual distortions—will clarify whether mechanisms of errant visual perception map onto dimensional clinical phenotypes of psychosis. Because SibIPDs do not have clinically significant psychotic symptomatology, their inclusion will allow some appraisal of the effect of treatment confounds on aberrant visual perception in psychosis. Eighth, the inclusion of 4- and 8-month follow-up appointments with IPDs will allow an examination of the associations between changes in psychotic symptomatology, and aberrant visual perception.

## APPROACH

### Overall Structure

This is an R01 currently in its third year. Dr. Scott Sponheim is the Principal Investigator. There are two sites: the Minneapolis VA Health Care System and the University of Minnesota’s Center for Magnetic Resonance Research (CMRR). Participants are recruited through the Minneapolis VA, and community-based providers in the Twin Cities area. Subjects complete psychophysical tasks, and undergo behavioral, clinical and cognitive assessment at the Minneapolis VA. Subjects undergo magneto-encephalography (MEG) scans while completing psychophysical tasks within the Brain Sciences Center at the VA. Participants undergo 7T MRI scanning at the CMRR, in addition to completing additional psychophysical tasks. In order to assess the role of altered visual perception in psychotic symptomatology, IPDs will have 4- and 8-month follow-up appointments for both MEG and MRI. Dr. Cheryl Olman oversees both 7T fMRI analyses, including retinotopic mapping of visual cortex and functional localization of other visual regions (e.g., LOC, ITC etc.), as well as the implementation of psychophysical tasks and neural network models. Dr. Seung Suk-Kang oversees both MEG analyses, and the derivation of cortical source signals using Ti-weighted structural scans. Dr. Nathaniel Helwig will oversee statistical analyses, and provide consultation on quantitative methods related to MRI and MEG analyses.

### Study Design

Total study enrollment will include 150 subjects total: 50 IPDs who are receiving outpatient or community-based mental health services, 50 SibIPDs, and 50 HCs. All participants will complete a clinical assessment protocol that will document current and lifetime symptomatology as well as perceptual illusions. For Aim 1, IPDs, SibIPDs, and HCs will complete visual perceptual tasks described below in addition to a brief standard cognitive battery to document overall intelligence and domains affected by psychotic disorders. For Aim 2 participants will complete separate sessions for MEG and MRI acquisitions. The 9 to 12 hour protocol will be conducted over a 2 to 3 day period, depending on participant factors. Clinical and conventional visual perceptual procedures will be interleaved as necessary in order to prevent ocular fatigue and to ensure the best data quality and motivation possible in participants, particularly IPDs. To assess within subject changes in visual distortions IPDs will additionally complete symptom ratings, MEG, and fMRI at 4- and 8-months after their baseline set of procedures.

### Interviews and Questionnaires

In order to assess symptom domains in IPDs, SibIPDs, and HCs, a trained and supervised research staff, a clinical psychology doctoral student, or a doctoral-level psychologist, completes the Structured Clinical Interview for DSM-IV (SCID-I; [46]) for each subject. To ensure comprehensive coverage of psychotic symptomatology (including insidious as opposed to rapid onset of psychosis) and to allow more comprehensive examination of dimensional clinical phenotypes, the Psychosis Module of the Diagnostic Interview for Genetic Studies (DIGS: Module K; [47]) is administered in place of the SCID Psychosis module. A trained research assistant conducts a medical chart review when records are available to obtain collateral information about the subject’s current level of functioning, and past and present symptomatology (which will be largely absent for SibIPDs and HCs). These records will also be used during a clinical consensus to finalize diagnoses.

Based on the SCID, chart information, study staff complete initial ratings of symptomatology using the Scale for the Assessment of Negative Symptoms (SANS: [48]) and the Scale for the Assessment of Positive Symptoms (SAPS; [49]), with additional queries built into the interviews to obtain the necessary information to complete all rating instruments. Global scores for negative (i.e., alogia, affective flattening, avolitionapathy, anhedonia-asociality, and attention) and positive (i.e., delusions, hallucinations, and positive formal thought disorder) symptom domains are then computed. Interviewers also make clinical ratings using the Brief Psychiatric Rating Scale 24-item version (BPRS; [50]) to quantify mood and other behavioral characteristics of clinical state.

Because the proposed work focuses on hallucinatory phenomena and includes individuals with intermediate levels of symptomatology (i.e., SibIPDs) we will also rely on dimensional measures that span from health to disorder (i.e., schizophrenia and other psychotic disorders). While current measures of psychotic symptomatology are derived from clinician-ratings (SAPS, SANS, BPRS), lifetime symptomatology is characterized with the Operational Criteria Checklist (OPCRIT; [51]) informed by interview and clinical records (again, largely absent for SibIPDs and HCs). For targeted assessment of hallucinatory phenomena, subjects complete the Structured Interview for Assessing Perceptual Anomalies (SIAPA; [9]) and questionnaires tapping visual illusions and distortions (i.e., the Schizotypal Personality Questionnaire (SPQ; [52]; and Personality Inventory for DSM 5 (PID-5; [53]) and sensory gating (SGI; [54]). Severity of visual hallucinations will be assessed via the BPRS, while the duration and temporal etiology of visual hallucinations will be assessed via the Psychosis module of the DIGS (i.e., K-DIGS). Other clinical measures including the SAPS, will allow us to distinguish the severity of visual hallucinations from other forms of hallucinations. Critically, the SIAPA, SGI. BPRS and SAPS/SANS are all assessed at each follow-up appointment for IPDs as well, allowing us to determine how shifting severity of aberrant visual phenomena is associated with changes in behavioral and neural findings during psychophysical tasks. Patient functioning is assessed using the Social Functioning Scale (SFS; [55]) and with the Global Assessment Scale (GAS; [56]).

Although the primary focus of the work is the dimensional assessment of psychotic symptomatology, diagnoses will secondarily be assigned according to DSM criteria through a consensus process involving at least two trained advanced degree clinical psychology students, or doctoral level psychologists, and include the review of a subject’s interview, symptom ratings, chart review, and informant information whenever possible. The diagnostic method will approximate the best-estimate approach as articulated by Leckman et al. [57]. Dr. Sponheim acts as the supervisor for all clinical assessments, and will oversee consensus ratings.

### Cognitive Assessments

We will include a small set of measures to assess general cognitive functioning and test for select deficits in IPDs and SibIPDs. Intellectual ability will be estimated from the Wide Range Achievement Test III [58], and performance on Matrix Reasoning and Similarities subtests of the Wechsler Adult Intelligence Scale-IV [59]. Additional cognitive tests will assess working memory (WAIS-IV Digit Span Forward and Backwards), episodic memory (California Verbal Learning Test-II [CVLT-II]; [60]), attention and set shifting (Trails A & B), processing speed (Digit Symbol), phonemic fluency (Controlled Oral Word Association Test (COWAT; [61]), and context processing (Dot Pattern Expectancy Task [DPX]; [62]).

### Aim 1

#### Psychophysical tasks

##### Location Masking Task:

The task has been described previously [22,63]. Briefly, participants first complete a procedure to determine the critical stimulus intensity (CSI) for the masking task to equate participants on the target threshold. After establishing a participant’s CSI, staff administer a target location task with a high-energy visual mask. A trial consists of a 300 ms fixation cross, 100 ms blank screen, 13 ms target, a variable post target period, and then a 26 ms mask. The participant identifies which quadrant the target appears in: the upper left, upper right, lower left, or lower right of fixation. The time between the onset of the target and the onset of the mask (SOA) varies between 0, 13, 27, 40, 53, 67, and 80 ms, with 12 trials per condition. Previous work has demonstrated that reduced accuracy on backwards location masking is reduced in patients with schizophrenia [64], differentiates schizophrenia from bipolar disorder [65], and that fragility in early visual percepts (i.e., reduced accuracy on SOAs 13 and 27) marks genetic liability specific to schizophrenia [22].

##### Degraded Stimulus Continuous Performance Test (DS-CPT):

For a detailed description of the task, please see Nuechterlein & Asarnow [66]. The task consists of a CPT with both task stimuli and background visually degraded: 40% of white numeral pixels are switched to black, and 40% of black background pixels switched to white. Sensory control trials are administered consisting of “just look” (participants instructed to look passively at the screen) and “press every” (participants instructed to respond to each stimulus) at 80 trials each. Following a practice block, subjects then receive DS-CPT instructions and complete three experimental blocks wherein 25% of stimuli are targets (“0”) while the remainder are nontargets (numerals “1” to “9”). Previous work has demonstrated that reduced perceptual sensitivity to target stimuli differentiates schizophrenia from bipolar disorder [67], and that first-degree relatives of patients with schizophrenia have a larger number of false alarms to stimuli that share contours with targets (numerals “6”, “8” and “9”), suggesting impaired contour detection in individuals with genetic liability for psychosis [68].

##### Broadband Surround Suppression:

**Figure 2.**
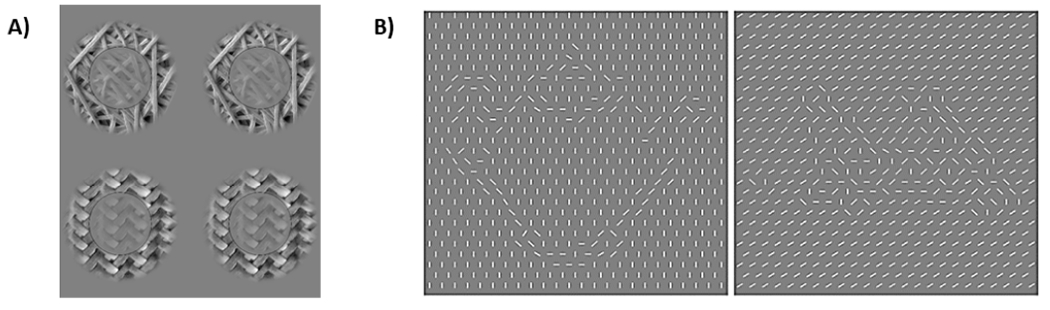
(**A**) Surround suppression in more naturalistic scenes will be studied with a 2AFC perceived contrast task at a single pedestal contrast (unsurrounded reference at 25% RMS contrast on one side of the screen, and target with 33% RMS contrast, matched or non-matched surround on the other side) for stimuli composed of line segments, natural scene segments, and synthetic textures (first and second-order statistics derived from natural scene segments, generated by Simoncelli’s “steerable pyramid” toolbox; [73]). This task will be used to quantify surround suppression in uniform and segmented textures. The same underlying model will be employed, but the more sparse neuronal responses will probe a wider range of neural network states for representations of the central stimulus, as well as constrain terms representing long-range projections signaling grouping probability or scene segmentation cues. (**B**). An object recognition task using line-segment textures to depict either meaningful or meaningless objects will let us test the effect of object recognition on contrast discrimination, fMRI and MEG responses in early visual cortex, and the interaction between neurophysiological signals in early visual cortex and prefrontal cortex.

In order to characterize basic visual functions in IPDs, SibIPDs, and HCs, we measure contextual modulation of perceived contrast of naturalistic textures (Figure 2A). Participants are instructed to compare texture disks with and without annular surrounds (while fixating on a central target and viewing both in peripheral vision) and indicate which of the texture disks has the greatest contrast. Instead of using traditional luminance gratings (consisting solely of regular stripes), broadband images (photographs of textures) are used. This is because the excitation/inhibition balance that regulates gain control is different for narrow band (grating) versus broadband (naturalistic texture) images [69]. In normal vision, detection of both contours and boundaries is improved in natural scenes because the texture similarity results in adaptive suppression [70]. By quantifying sensitivity to the relative orientation of center and surround, which changes the predictive power of the surround, we can test the hypothesis that predictive coding mechanisms that regulate local gain control are altered for IPDs and SibIPDs. An additional manipulation of attention during fMRI and MEG scans will allow us to discern the differential impact of focal and radial attention respectively [71,72].

##### Fragmented Ambiguous Object Task (FAOT):

The development of the FAOT is detailed in Olman et al. [74]. The task stimuli equate low-level features such as orientation and contrast, while simultaneously manipulating intermediate- and higher-level features (i.e., contour organization and contour shape; Figure 2B). The task is better suited to tapping higher-level object recognition and corresponding neural correlates than the DS-CPT; previous studies have demonstrated that visual areas such as inferior temporal cortex (ITC; [75]), and medial frontal gyrus (MFG; [76]) are implicated in object recognition. One-third of FAOT stimuli are highly recognizable (designated “meaningful”), one-third are moderately recognizable (designated “ambiguous”), and one-third are minimally recognizable (designated “meaningless”). During the FAOT a participant determines whether they recognize the object (“yes”/“no”). Catch trials of objects deemed to be highly recognizable are also included as an attentional control. The FAOT assesses participants’ abilities to distinguish meaningful shapes from noise, and permits examination of high-level long-range mechanisms required for integrating global scene information with local features such as orientation contrast. We expect that a failure of high-level long-range effects will be evident in behavioral data as increased reaction times for detecting objects, and that both IPDs and SibIPDs will have impaired object recognition and neural responses to stimuli relative to HC.

#### Statistical analyses

In addition to interpreting the results of long-range and local influences on alterations in visual perception by utilizing the proposed computational model (Aim 3). we will use data-driven nonparametric regression to examine relationships between self-reported visual hallucinations/ alterations and psychophysical task performance [77–79]. Nonparametric regression models the relationship between the predictor and response variables based on the data (e.g., they are not assumed to be linear or quadratic; [80]). The relation of self-reported experience of visual misperceptions to task performance can be formulated as:

y=(contrast, scene statistics, object identity)+ e

where *y* is self-reported visual distortions (e.g., hallucinations), “contrast” represents the performance on BBSS, and both scene statistics (i.e., contour organization), and “object identity” (i.e., contour shape) are indicative of performance on FAOT. f() is the unknown function linking the perceptual task performance to the self-reported visual hallucinations and distortions—the function estimated from the data—and e is the model error term. Using these methods, we can determine how individual and group differences in self-reported visual misperceptions relate to differences in laboratory-based tasks without *a priori* assumptions about the nature of the relationship. We will complement these analyses with the Simultaneous Component Analysis (SCA) model, which will allow us to capture both intraindividual and interindividual differences across numerous observations (i.e., baseline, 4-month, and 8-month follow-up appointments; [81]).

### Aim 2

#### Functional neuroimaging

##### MEG Acquisition:

MEG data is being collected at 1017 Hz using a 248-sensor axial gradiometer MEG system (Magnes 3600WH, 4D-Neuroimaging), located within an electromagnetically-shielded room in the Brain Sciences Center at the Minneapolis VA Medical Center. Environmental noise will be removed from the data by relating signals from separate reference channels to cortical channels. Eye movements are at the same sampling rate using horizontal and vertical electrooculograms (HEOG, VEOG) for later artifact removal. Sensor configuration and head position are obtained using a spatial digitizer. The MEG is equipped with a calibrated projector and accompanying screen to present visual stimuli.

##### MEG Processing:

MEG data processing and analysis procedures will be conducted using custom Matlab scripts (The MathWorks, Inc. Natick, MA, USA). In order to remove slow artifacts related to slow-drift noise, high-pass filtering with a cut-off of .1 Hz will be applied to the data. MEG signals will be segmented into trial periods defined in relation to task timing and trial parameters (e.g., stimulus onset). Independent component analysis (ICA) using the Fast ICA algorithm in a custom pipeline will be used to remove additional signal artifacts [82]. Noisy sensors and epochs with artifacts due to movement will be identified and removed with analyses of the low-(<8 Hz) and high-frequency (>30 Hz) signal powers and visual inspection. These epochs will be rejected before initiation of ICA decomposition procedures.

In order to obtain an optimal ICA decomposition and prevent underfitting or over-fitting of the 248-sensor data, dimensionality will be estimated using the Bayesian information criterion (BIC; [83]). Principal component analysis (PCA) will be applied to reduce data to the estimated number of dimensions before applying ICA. Along with Independent components (ICs) reflecting artifacts such as vertical and horizontal eye-movement and heart signals, other possible sources of noise in beta and gamma frequency oscillations will be considered, including microsaccades [84], and small muscle contractions of the neck and head. Myogenic and microsaccade artifacts will be identified based on characteristics of IC topography, spectral power, time-series, and the relationship with EOG sensor signals, which have been described in McMenamin et al. [85], and Keren et al [86]. Artifact ICs will be removed from MEG data prior to reconstituting the signals.

##### Derivation of Cortical Source Signals:

**Figure 3.**
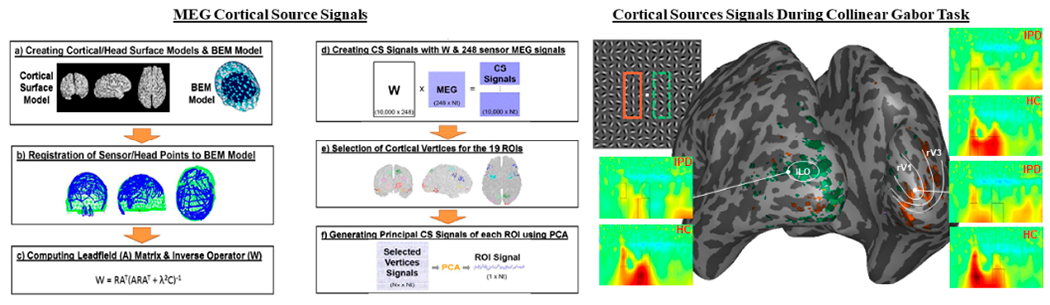
**Left.** Computation of cortical source signals. 248 channel axial gradiometer MEG recordings during the Gabor contour task were localized to cortical ROIs. **Right.** Example of cortical source signals applied to a Collinear Gabor Task. Using a retinotopic mapping technique during fMRI, early visual cortical areas were mapped according to their functional and spatial layout. This allowed us to map regions of early visual cortex where the contour appeared in the visual field. These are shown on the inflated 3D brain for V1 and V3 (orange), and ILO (green).___IPDs have reduced power in ILO, as well as right V1 and V3.

Procedures to compute cortical source signals are summarized in the left panel of Figure 3. Briefly, they consist of the following steps: (1) FreeSurfer is used to create individualized cortical surface models with T1-weighted structural MRI data; (2) Cortical surface models with more than 100,000 cortical vertices for each participant are down-sampled to generate an epi-cortical surface model for each participant with approximately 10,000 cortical vertices; (3) The boundary element method (BEM; [87]) model with 3 layers (scalp, outer skull, and inner skull), 1,200 elements, and 600 nodes is applied for the forward calculation of magnetic field; and 4) Coordinates of sensor locations and head surface data collected during the MEG session are registered to the cortical surface and BEM models generated from MRI. Registration in MEG is done using coordinate rotation by linear transformation and translation based on 5 landmarks: nasion, left/right pre-auricular points and VEOG and MEOG coils on the forehead. Pictures are taken of each participants ears, so that that these registration points can be applied to structural data.

To solve the inverse problem as part of computing source signals, the leadfield matrix relating sensor space (MEG) to cortical source space (fMRI) will be created using the BrainStorm program. The inverse operator W (center panel Figure 3) is derived from the leadfield matrix, source/noise covariance matrices, and regularization parameter determined by using a signal-to-noise ratio (SNR) estimate. The inverse operator allows direct conversion of 248 MEG sensor signals to the 10,000 cortical vertex signals. To create cortical source time-series for active brain areas we will identify regions by employ functional localizers in retinotopic ally mapped regions of striate cortex [88], task contrasts in high-level visual regions, and significant PPI in frontal (potentially parietal) regions. Typically, 40 to 80 vertices are selected for each cortical ROI. In order to generate a principal signal of an ROI from the multiple vertex signals PCA will used to define the first dominant component be carried out for vertex signals belonging to each ROI, with the first PCA selected as the representative signal of the ROI. An example of cortical source signals applied to a contour detection task can be seen in the right panel of Figure 3.

##### fMRI Acquisition:

fMRI experiments will be performed using a 7 Tesla scanner at the CMRR. Briefly, a Nova Medical single channel (circularly polarized) transmit, 32-channel receive head coil is used to acquire functional data. Anatomical reference data will be acquired in a separate scanning session at 3 Tesla. The scanner is equipped with a calibrated projector for display of visual stimuli and MR-compatible button boxes for subject response collection. Eye-tracking equipment is available and are used to verify fixation stability. Functional data are acquired with 2 s temporal resolution and 1.6 mm isotropic spatial resolution, using multiband and parallel imaging strategies to accelerate acquisition in the through-slice as well as phase-encode direction [89]. PE-reversed EPI scans are acquired to enable distortion compensation during post-processing.

##### fMRI Processing:

fMRI data processing will be performed using a combination of AFNI (preprocessing, GLM and functional/anatomical registration), and FreeSurfer (surface-based visualization and cortical segmentation). The majority of the analysis will focus on ROI-based analyses in predetermined ROIs in visual cortex (e.g., V1, V2), in order to mitigate the multiple comparisons problem. Posterior ROIs (in retinotopically mapped areas of visual cortex; top panel Figure 3) will be defined by utilizing functional localizers. Data in these regions will be extracted and averaged to estimate response amplitudes in each visual area. Additional regions in higher-level visual areas (e.g., ITC, MFG) will be defined using whole-brain analysis during BBSS (attended and unattended texture patches) and FAOT (meaningful vs. meaningless stimuli). Voxel-byvoxel contrast maps for the entire brain will be calculated. Individual voxels correlated with the task at uncorrected p < 0.01 will be subjected to cluster-size thresholding in order to select regions related to the task with corrected *p* < 0.01. These regions of interest will be seeds for task-dependent connectivity (psychophysiological interaction) analyses (PPI; [90]) to assess the role of downstream visual areas in temporal and frontal cortices.

##### Computation of time-frequency (TF) energy and phase synchrony:

Time-domain analysis reliably delineates neural responses that are consistently timed with a stimulus. To account for the possibility that individuals with psychosis show variability in evoked activity, we will characterize changes in frequency across time. We will use a RID algorithm to resolve time-frequency elements in EEG signals. Dr. Kang has expertise in generating RID waveforms.

#### Statistical analyses

Statistical tests of TF energy for MEG-derived cortical source signals of functionally localized ROIs will be carried out to describe neural activity associated with regulation of early visual processes that to the formation of visual percepts, and to test whether anomalous neural responses evident in IPDs and SibIPDs predict self-reported visual hallucinations and illusions. In order to ensure that we capture effects not predicted by the proposed computational model in Aim 3, we will use nonparametric mixed-effects (NPME; [91,92]) regression to examine functional relationships between self-reported experiences of visual misperceptions and abnormalities in the activity or interactions of visual cortex with other brain regions. NPME models are an extension of a linear mixed-effects regression (LMER; [93]) model. LMER models correlated data (e.g., repeated measures). Unlike LMER models, a NPME model does not require a priori assumptions about the nature of the relationship between the response (e.g., self-reported visual misperceptions) and the predictors (responses and interactions of brain regions).

### Aim 3

#### Computational model

The model that will be used to interpret behavioral and neuroimaging data is adapted from one developed by Schwartz and colleagues, [39]. It has two main components that describe center/surround interaction in primary visual cortex: divisive normalization and segmentation. The former is implemented by local inhibitory (i.e., GABAergic) neurons [36] with regulation from excitatory neurons that are modulated by attention [94]. During both attention and object-recognition manipulations, the local GABAergic gain control is regulated by long-range influences that are nearly always mediated by Glutamatergic neurons [95,96]. Crucially, the strength of this regulation is sensitive to natural scene statistics; this ‘flexible normalization [97] removes suppression at probable object boundaries, leading to efficient scene segmentation [98].

Neuronal responses in primary visual cortex are estimated as a mixture of two separate neuronal populations, one of which experiences surround suppression (*E*_*g*|*c,s*_) and one that does not (*E*_*g*|*c*_):

$$E_{i}=(1-p)E_{g|c}+pE_{g|c,s}$$

where *E_i_* is the channel (orientation column) tuned to the *i*^th^ orientation, and *p* is the probability that the classical receptive field and the extraclassical receptive field belong to the same object. Thus, the p term reflects learned scene statistics encoded by extrastriate neuronal populations [99] that regulate surround suppression in VI. Using this model to simultaneously fit data from multiple behavioral and neuroimaging tasks will allow an estimate of the strength and tuning of the *p* parameter as well as the gain control terms that determine *E*_*g*|*c,s*_, (early visual responses after surround suppression) and *E*_*g*|*c*_ (responses in the subset of neurons in early visual cortex that are not subject to surround suppression).

##### Application of Computational Model:

The tasks in Aim 1 and Aim 2 were selected because of their ability to quantify the separate contributions of visual mechanisms to perception. These tasks will allow examination of how early visual mechanisms may be regulated by higher-order neural computations supporting spatial attention, scene segmentation and object recognition.

Two parameters regulate the local response term (*E*_*g*|*c,s*_): a scalar that reflects the size of the extra-classical receptive field, and a scalar that reflects the relative strength of local inhibition. Three additional parameters regulate the grouping probability (*p*): a term that represents sensitivity to relative orientation, a term that represents sensitivity to higher-order scene statistics, and a term that represents object detection. The model can be used to predict 7 experimental outcomes: suppression of perceived contrast by parallel and orthogonal similar and dissimilar textures (4), fMRI response suppression by parallel vs. orthogonal similar vs. dissimilar textures (2), and VI response modulation by object recognition (1).

Preliminary analyses of fMRI data show a significant overall VI signal increase during viewing of meaningful compared to meaningless objects (FAOT), and task-dependent connectivity analysis has revealed that IPD and HC differ in the relative strength of the interaction between VI and pre-frontal cortex. Thus, we will also correlate task-dependent connectivity measures from the two tasks against appropriate components of the long-range connectivity term (p) in the model to verify model estimates of the strength of the long-range grouping terms, to test the hypothesis that alterations in long-range (excitatory) neural mechanisms are altered in SibIPDs.

#### Statistical analyses

Classical multivariate statistics (MANOVA) will be used to assess group differences in relative fit of the model parameters. Group by term interactions of interest involve parameters *E*_*g*|*c,s*_ and *E*_*g*|*c*_ based on our previous findings that differences in orientation tuning of surround suppression are evident in IPDs [14]. We will also use the modeled neural population responses to develop a practical estimate for the effective contrast that enables systematic comparison across different stimulus categories (e.g., sinusoidal luminance modulation, naturalistic textures and natural scene segments). The hypothesized elevation of perceived contrast in naturalistic stimuli, combined with altered top-down constraints will provide a mechanistic explanation for hallucinations and visual alterations.

### Sample Size Justification

Preliminary studies provide data from which to estimate required sample sizes. For behavioral tasks in Aim 1, existing data on surround suppression indicate a moderate mean effect size (Cohen’s *d* = 0.66). From this, we estimate that group sizes of 38 with α = 0.05 and power = 0.8 would be sufficient to detect effects employing 2-tailed tests. Considering multiple regression analyses will be extensively used, the observed associations in pilot data for Aim 2 (0.09 < *R*^2^ < 0.49) were used to estimate sample size. Given that *f*^2^=R^2^/(1 – *R*^2^) [100], sample sizes between 14 and 109 (for α =0.05 and power = 0.90) are required. Thus, a total of 150 individuals studied across the three groups will ensure sufficient power to detect effects.

## SUMMARY

The precise etiology of hallucinations and visual distortions in psychosis is unknown. By combining psychophysical tasks, functional neuroimaging, and a computational model, this project has the potential to yield a mechanistic understanding of visual hallucinations and distortions in subjective visual experience seen across the psychosis spectrum. By including first-degree relatives, we can determine if more subtle alterations in visual experience specifically reflect genetic contributions. As a result, this study will allow ascertainment of how visual perception varies across dimensional phenotypes of psychosis noted in schizophrenia spectrum disorders and bipolar affective disorder. The knowledge derived from this research will facilitate an understanding of neural mechanisms that give rise to psychotic symptoms, and the associated functional impairments of the disorder. Our hope is that the results can be used to formulate novel targeted treatments for psychosis, develop screening tools to help predict who is likely to convert to a psychotic disorder, and guide attempts to mitigate the risk for conversion, thereby reducing the prevalence and substantial disease burden of psychosis on society.
